# Matrix Metalloproteinases Repress Hypertrophic Growth in Cardiac Myocytes

**DOI:** 10.1007/s10557-020-07138-y

**Published:** 2021-01-05

**Authors:** Gerhild Euler, Fabian Locquet, Joanna Kociszewska, Yvonne Osygus, Jacqueline Heger, Rolf Schreckenberg, Klaus-Dieter Schlüter, Éva Kenyeres, Tamara Szabados, Péter Bencsik, Péter Ferdinandy, Rainer Schulz

**Affiliations:** 1 Institute of Physiology, Justus-Liebig-Universität Giessen, Aulweg 129, 35392 Giessen, Germany; 2 Department of Pharmacology and Pharmacotherapy, University of Szeged, Szeged, Hungary; 3 Pharmahungary Group, Szeged, Hungary; 4 Department of Pharmacology and Pharmacotherapy, Semmelweis University, Budapest, Hungary

**Keywords:** Cardiomyocytes, Hypertrophy, Matrix metalloproteinase, Signaling kinases

## Abstract

**Purpose:**

Matrix metalloproteinases (MMPs) are identified as modulators of the extracellular matrix in heart failure progression. However, evidence for intracellular effects of MMPs is emerging. Pro- and anti-hypertrophic cardiac effects are described. This may be due to the various sources of different MMPs in the heart tissue. Therefore, the aim of the present study was to determine the role of MMPs in hypertrophic growth of isolated rat ventricular cardiac myocytes.

**Methods:**

Cardiomyocytes were isolated form ventricular tissues of the rat hearts by collagenase perfusion. RT-qPCR, western blots, and zymography were used for expression and MMP activity analysis. Cross-sectional area and the rate of protein synthesis were determined as parameters for hypertrophic growth.

**Results:**

MMP-1, MMP-2, MMP-3, MMP-9 and MMP-14 mRNAs were detected in cardiomyocytes, and protein expression of MMP-2, MMP-9, and MMP-14 was identified. Hypertrophic stimulation of cardiomyocytes did not enhance, but interestingly decreased expression of MMPs, indicating that downregulation of MMPs may promote hypertrophic growth. Indeed, the nonselective MMP inhibitors TAPI-0 or TIMP2 and the MMP-2-selective ARP-100 enhanced hypertrophic growth. Furthermore, TAPI-0 increased phosphorylation and thus activation of extracellular signaling kinase (ERK) and Akt (protein kinase B), as well as inhibition of glycogen synthase 3β (GSK3β). Abrogation of MEK/ERK- or phosphatidylinositol-3-kinase(PI3K)/Akt/GSK3β-signaling with PD98059 or LY290042, respectively, inhibited hypertrophic growth under TAPI-0.

**Conclusion:**

MMPs’ inhibition promotes hypertrophic growth in cardiomyocytes in vitro. Therefore, MMPs in the healthy heart may be important players to repress cardiac hypertrophy.

## Introduction

Heart failure is one of the major causes of death in industrial countries and cardiac hypertrophy is a prerequisite of heart failure development. Cardiac hypertrophy is a multifactorial disease that can be caused by enlargement of the cardiomyocytes themselves. But also proliferation of cardiac fibroblasts [1] or the assembly of amyloid fibrils in the extracellular space [2] contribute to the enlargement of the heart, stiffening of the wall chambers, and finally impaired cardiac function.

Many therapeutics have been defined already in order to treat cardiac hypertrophy and to prevent heart failure progression, i.e., inhibitors of the renin-angiotensin system or calcium channel blockers. However, not all patients respond to these drugs, or only to a limited extent [3]. Therefore, identification of new therapeutic targets in the treatment of cardiac hypertrophy is urgently needed. In this study, we focus on the role of matrix metalloproteinases (MMPs) in cardiac hypertrophy.

During cardiac remodeling, processes that include cardiac hypertrophy and restructuring of the extracellular matrix are induced. MMPs are found activated and involved in cardiac remodeling. Primarily, these enzymes were described to degrade the extracellular matrix and, thereby, contribute to matrix remodeling. However, recent research demonstrated induction of intracellular processes in cardiomyocytes by MMPs, either due to proteolytical processes at diverse cell surface ligands, receptors, or signaling molecules, or inside the cell, since MMPs are not only restricted to the extracellular matrix but are also active inside the cardiomyocytes [4]. Going along with these findings, an increasing number of substrates that can be targeted by MMPs are defined, and the role of MMPs in diverse processes of cardiac remodeling is steadily increasing. Numerous studies demonstrate the influence of MMPs on cardiac hypertrophy, although with different outcomes [5–8], e.g., reduction or induction of cardiac hypertrophy by MMPs. Since most of these studies were conducted in in vivo animal models, often in transgenic mice, the divergent outcomes may be due to the complexity of MMP functions in different cell types.

Currently, around 30 MMP members are known. They are proteolytic enzymes that are synthesized as inactive pro-forms which are activated upon their release from different cell types into the extracellular matrix [9]. Also in the heart, the role of MMPs was primarily described to enhance extracellular matrix degradation and fibrosis, i.e., in ischemic-reperfused myocardium [10, 11] or in myocarditis [12]. However, the spectrum of cardiac MMP targets is currently expanding. Under angiotensin II (AngII) infusion in MMP-2 knockout mice, cardiac hypertrophy progressed earlier and with greater severity, thereby indicating a protective role of MMP-2 in progression of cardiac hypertrophy [5]. Others identified MMP-7 as a mediator of hypertrophy under AngII infusion [6]. Furthermore, macrophage-specific overexpression of MMP-9 in transgenic mice enhanced cardiac hypertrophy [7]. These findings indicate the complex intercellular interaction of MMPs. In addition to MMPs, ADAMs (A disintegrin and metalloproteinases), which are closely related to the MMP family, have pro- and anti-hypertrophic actions [13]. Both, MMPs and ADAMs, can influence hypertrophic signaling cascades like Akt (protein kinase B) or ERK (extracellular signal-regulated kinase) via extracellular cleavage of cell surface integrins or receptors.

The current study focused on MMP effects on cardiomyocytes independent of other cell types in the heart. We analyzed the expression patterns of MMPs in isolated ventricular cardiomyocytes of adult rat and demonstrate pro-hypertrophic effects under MMP inhibition. With this, we identified MMPs as repressors of cardiomyocyte hypertrophy.

## Materials and Methods

### Materials

Medium 199 was obtained from Boehringer (Mannheim, Germany), fetal calf serum from PAA (Linz, Austria), crude collagenase from Biochrom (Berlin, Germany), and MMP inhibitors from Merck (Darmstadt, Germany). Antibodies for MMP-2, MMP-9, and MMP-14 and integrin1β were from Abcam, antibodies against ERK and p-ERK were from Santa Cruz (Heidelberg, Germany) and against p-GSK3β were from Cell Signaling (Frankfurt, Germany), and vinculin antibodies were from Sigma-Aldrich (Taufkirchen, Germany). Secondary antibodies were from Cell Signaling (Frankfurt, Germany). GSK-3β Activity Assay Kit was from Sigma.

### Cell Isolation and Cardiomyocyte Cultures

**Fig. 1:**
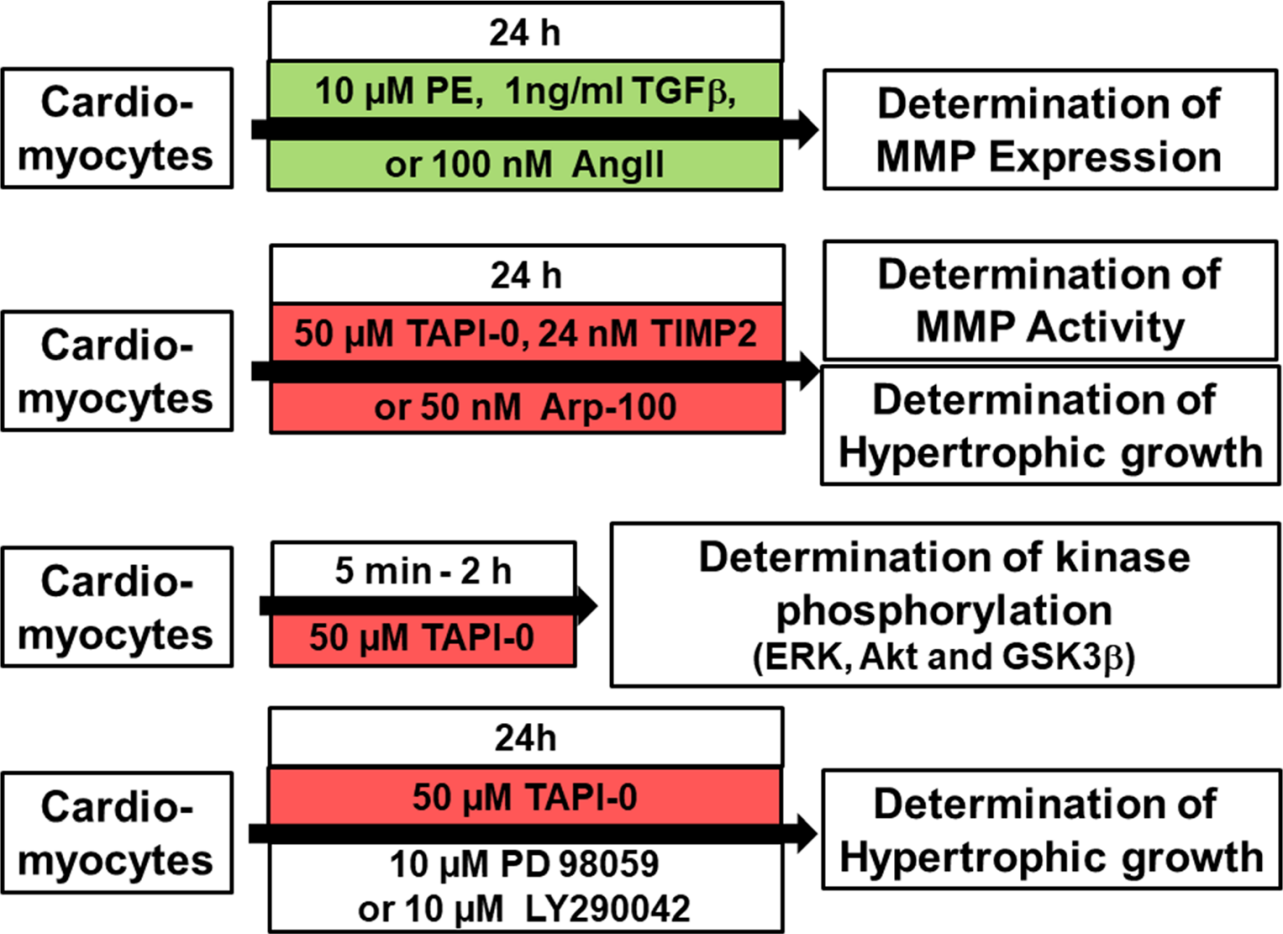
Study design and treatment protocol. Cardiomyocytes were treated with hypertrophic growth stimuli (indicated in green) for 24 h in order to determine MMP expression under these stimuli. In another set of experiments, cardiomyocytes were treated with MMP inhibitors (indicated in red) for 24 h in order to determine their influence on hypertrophic growth. In addition, inhibition of MMP activity was proved in zymography assays. Finally, phosphorylation of kinases, typically related to hypertrophic growth (ERK, Akt, and GSK3β), was determined under MMP inhibition with TAPI-0 within 5 min to 2 h, and the influence of ERK or PI3K inhibition on TAPI-0 induced hypertrophic growth was determined

Ventricular cardiomyocytes were isolated from 200 to 250 g male Wistar rats, suspended in basal culture medium and plated on culture dishes, which were preincubated overnight with 4% fetal calf serum in medium 199, as previously described [14]. The basal culture medium (CCT) was modified medium 199 including Earle’s salts, 2 mM L-carnitine, 5 mM taurine, 100 IU/ml penicillin, 100 μg/ml streptomycin, and 10 μM cytosine-β-D-arabinofuranoside (pH 7.4). Three hours after plating, the dishes were washed twice with CCT medium. This results in cultures of about 90% quiescent rod-shaped cells on average. Cardiomyocytes were treated as depicted in Fig. 1.

### Cardiac Specimens from Spontaneous Hypertensive Rats

Cardiac specimens originate from a study published by Schreckenberg et al. [15]. Female SHR rats and normotensive Wistar rats were kept over 7.5 months. At the age of 1.5 and 7.5 months, blood pressure was determined by tail-cuff measurements. At the end of the experimental period, rats were anesthetized by isoflurane inhalation. After cervical dislocation, the hearts were isolated and perfused in Langendorff technique to remove blood contamination. Heart weight to tibia length was determined as parameter of cardiac hypertrophy. The hears were frozen in liquid nitrogen and stored at − 80 °C.

### Real-Time RT-PCR

Total RNA from cardiomyocytes was extracted with Trizol (Invitrogen) as described by the manufacturer. This was followed by DNAse treatment and reverse transcription with QuantiTect Reverse Transcription Kit from Qiagen. For each assayed gene, annealing temperature and the number of cycles resulting in a linear amplification range were tested. RT-PCR was performed in an automated thermal cycler and detected with the Bio-Rad detection system (Bio-Rad) using SYBR Green fluorescence for quantification. The calculations of the results were carried out according to the 2^−ΔΔCt^ methods as described [16]. Gene expression was related to β2-microglobulin (B2M) as housekeeping gene. Primer sequences are listed in Table 1.

**Table 1:** Primer sequences for real-time RT-PCR

Gene	Forward primer	Reverse primer
MMP-1	GCCACTCCCTTGGACTCACTCA	TGCACCTGTTGGCTGGATGGGA
MMP-2	TACAGACGGCTACCGCTGGTGT	AGCGCTGGTGCAGCTCTCAT
MMP-3	ACAGACCTGGCCCGTTTCCA	GCAGGGTGCTGACTGCATCGAA
MMP-7	AGGCTCACCCTGTTCCGCAT	TCCACTGCACTGGTGGCCTT
MMP-8	TGCAGCGCTTCTTCGGCTTG	TTGGGGCTTCCCGGAGTTAGCA
MMP-9	TCACGGACACACAGCTGGCA	ACCACAGCGCGGTGAACGAA
MMP-13	TGCCCATGAGCTTGGCCACT	TTGGGGTGCTTAGGGTTGGGGT
MMP-14	AGCAGCAACTTCAGCCCCGA	TCCGAATCGGCCTTGCCTGT
Integrin	TTGTGGAGACTCCAGACTGTCCTACT	TCATTTTCCCTCATACTTCGGATT
B2M	CCAGCGTCGTGATTAGCGAT	CAAGTCTTTCAGTCCTGTCC

Primers are listed in 5′ to 3′ direction

### Immunoblotting

At the end of the treatment, cells were washed twice with ice-cold phosphate-buffered saline (PBS) before being lyzed on ice in RIPA buffer [50 mmol/L Tris/HCl, pH 7.5, 150 mmol/L NaCl, 1% Nonidet P-40, 0.5% deoxycholat, 0.1% sodium dodecylsulfate (SDS)] containing protease inhibitor [1 mmol/L phenylmethylsulfonylfluoride (PMSF), 1 mmol/L ethylenediaminetetraacetic acid (EDTA), 1 mg/L pepstatin]. The protein content was determined by Lowry protein assay [17]. For control, every blot was stained with Ponceau Red before blotting. Primary antibodies were used according to the manufacturer’s instructions. Protein bands were detected by horseradish peroxidase-labeled anti-rabbit antibodies by the use of the ECL detection system. Vinculin was used for loading controls.

### Zymography Assay

Gelatinolytic activity of MMP was examined as previously described in detail [18, 19]. Briefly, 8% polyacrylamide gels, co-polymerized with gelatin (2 mg/ml), were loaded with 50 μg of protein. After electrophoresis, gels were washed with renaturation buffer (containing 2.5% Triton X-100), and then incubated in development buffer to eliminate Triton X-100. Gels were stained with 0.05% Coomassie Brilliant Blue, and gelatinolytic activities were detected as transparent bands against the dark-blue background. Band intensities were quantified (Quantity One software, Bio-Rad, Hercules, CA) and expressed in arbitrary units.

### Incorporation of ^14^C-Phenylalanine

To determine the rate of protein synthesis, incorporation of phenylalanine was measured by exposing cultures to L-^14^C-phenylalanine (0.1 μCi/ml) for 24 h. Incorporation of radioactivity into acid-insoluble cell mass was determined as described before [20].

### Cross-Sectional Area

The cardiomyocyte size was determined on micrographs digitalized by a charge-coupled device camera, as described elsewhere [21]. Five micrographs were taken randomly per sample, and all rod-shaped myocytes in these fields were measured. A total of about 30 cells were measured for one condition in one preparation. The width/diameter of cardiomyocytes was determined at the widest point of each cardiomyocyte using the software program Analysis from SIS. The cross-sectional area of cardiomyocytes was calculated by the following formula: (radius)^2^ × π.

### Determination of GSK-3β Activity

The GSK-3β activity was determined by the use of GSK3β activity assay kit from Sigma. The total protein was extracted as described for immunoblots. Then GSK3β was immunoprecipitated. After addition of a peptide substrate and γP^32^-ATP, labeling of the peptide by GSK-3β was determined.

### Statistics

Data are given as mean ± standard deviation from *n* different animals. Statistical comparisons were performed by ANOVA (one-way or two-way analysis of variance) and Student-Newman-Keuls test or Student *t* test. A *p* value of less than 0.05 was considered statistically significant.

## Results

### Expression of MMPs in Cardiomyocytes

**Fig. 2:**
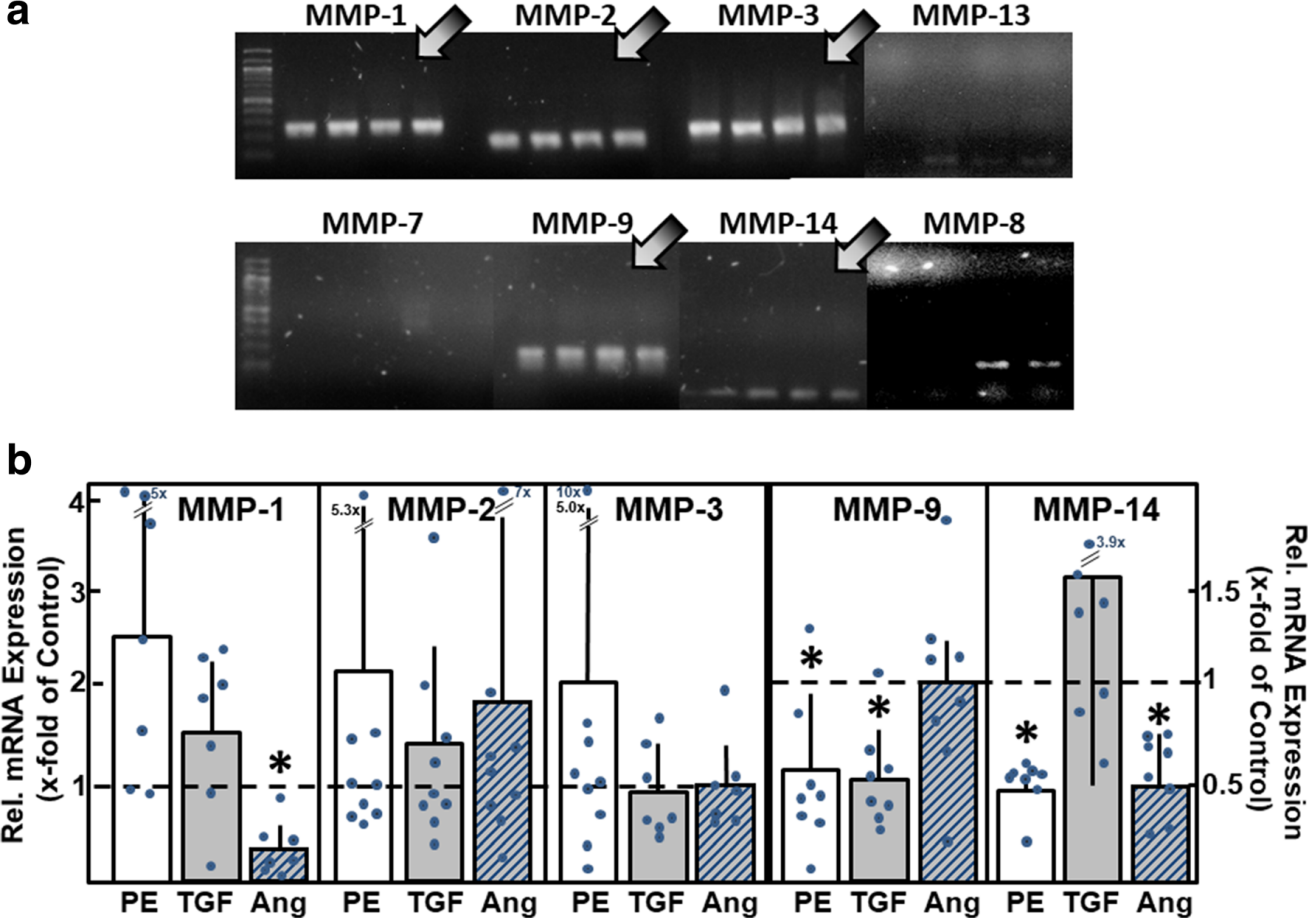
MMP mRNA expression in cardiomyocytes. (A) Total RNA was isolated from ventricular cardiomyocytes, RT-PCR performed, and amplification products were loaded on agarose gels (*n* = 4 independent culture preparations). mRNA expression of MMP types1, 2, 3, 9, and 14 was detected (indicated by arrows). (B) To determine the changes in mRNA expression under hypertrophic growth stimulation, cardiomyocytes were incubated for 24 h with phenylephrine (PE, 10 μM), TGFβ_1_ (1 ng/ml), or angiotensin II (Ang, 100 nM). mRNA expression of MMP types was analyzed by real-time RT-PCR (*n* = 7 – 9, *p < 0.05 vs. controls)

As reviewed by Spinale [22], eight different MMPs were identified in the whole myocardium, namely, MMP-1, MMP-2, MMP-3, MMP-7, MMP-8, MMP-9, MMP-13, and MMP-14. We now analyzed mRNA expression of these MMPs on the cellular level in isolated adult rat cardiac myocytes. As depicted in Fig. 2A, reproducible and clear bands were detected for MMP-1, MMP-2, MMP-3, MMP-9, and MMP-14, thereby indicating that these MMPs are expressed in cardiomyocytes under basal culture conditions. A weak and non-reproducible mRNA expression was found for MMP-8 and MMP-13, whereas the expression of MMP-7 was not detected at all.

To determine if this pattern of MMP mRNA expression changes under hypertrophic growth stimulation, cardiomyocytes were stimulated with the α-adrenoceptor agonist phenylephrine (PE, 10 μM), with transforming growth factor β1 (TGFβ1, 1 ng/ml), or angiotensin II (AngII, 100 nM) for 24 h. PE and AngII treatments lead to well-known pro-hypertrophic effects in cardiomyocytes [23–26], whereas TGFβ1 is known to induce hypertrophic responsibility in cardiomyocytes and cardiac remodeling in the heart [27, 28]. Interestingly, MMP upregulation was not found under hypertrophic growth stimulation. Instead, some MMP mRNAs were downregulated under pro-hypertrophic conditions: PE reduced MMP-9 mRNA to 0.56 ± 0.36 times of control, and MMP-14 mRNA to 0.48 ± 0.15 times of control (*n* = 8, *p* < 0.05). AngII stimulation reduced the level of MMP-1 mRNA to 0.29 ± 0.28 times of control and MMP-14 mRNA to 0.50 ± 0.20 times of control (*n* = 7 – 8, *p* < 0.05). TGFβ1 reduced mRNA expression of MMP-9 to 0.52 ± 0.25 times of control (*n* = 8, *p* < 0.05) (Fig. 2B).

**Fig. 3:**
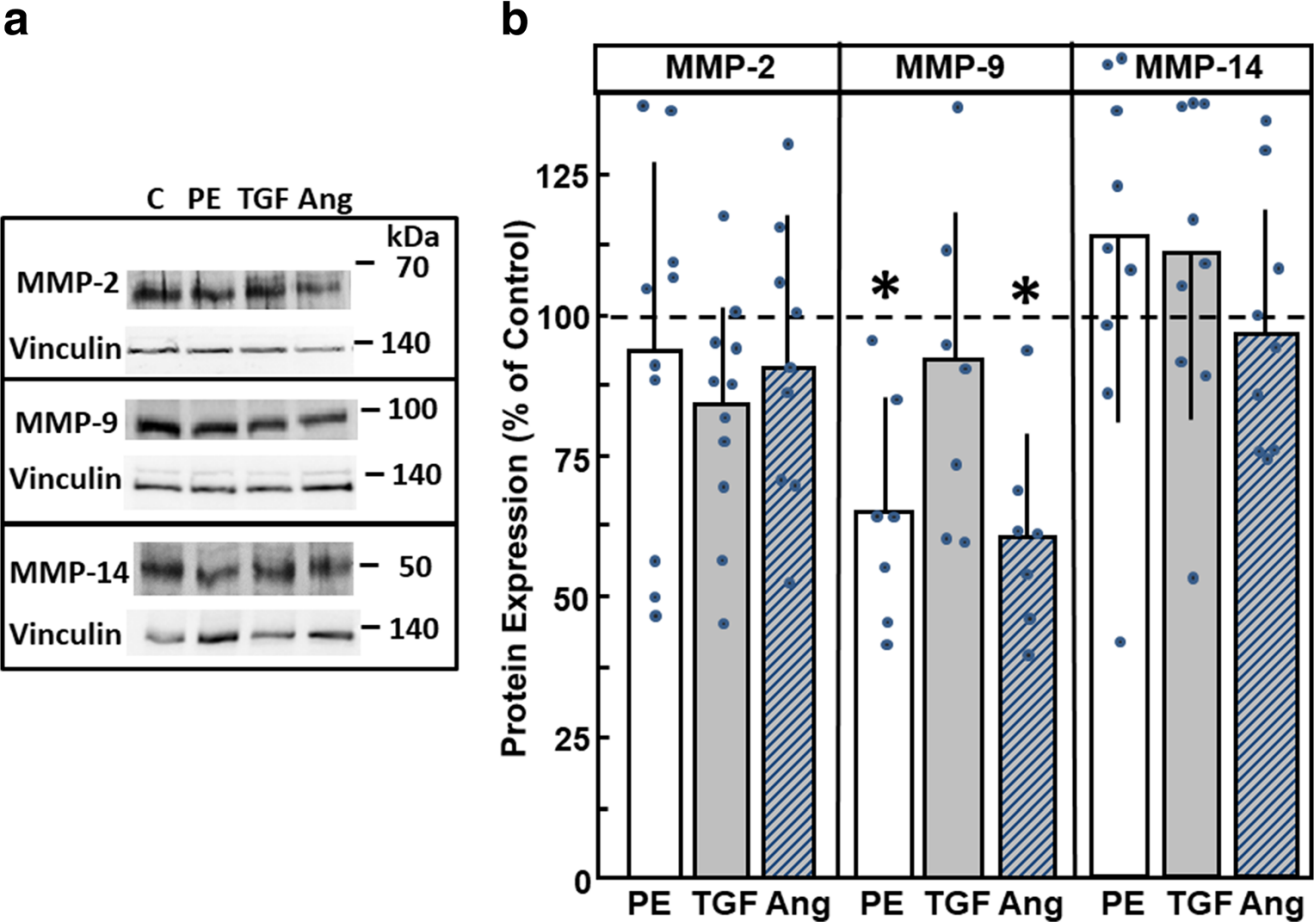
MMP protein expression under hypertrophic growth stimulation. Cardiomyocytes were incubated for 24 h with phenylephrine (PE, 10 μM), TGFβ_1_ (1 ng/ml), or angiotensin II (Ang, 100 nM). Protein expression of MMP-2, MMP-9, and MMP-14 could be detected in (A) Western blots, and (B) bands were densiometrically evaluated. Vinculin expression served as loading control. (*n* = 7 – 11, *p < 0.05 vs. controls)

Furthermore, we tested if reduced MMP mRNA expressions under hypertrophic stimulation are reflected on the protein levels. Therefore, cardiomyocytes were again stimulated with PE, AngII, and TGFβ1. As depicted in Fig. 3A, protein expressions of MMP-2, with a main band at 68 kDa, of MMP-9 (at 92 kDa), and MMP-14 (around 50 kDa) were detected. Quantification of Western blots revealed no significant upregulation of MMPs. However, MMP-9 expression declined under stimulation with PE or AngII (Fig. 3B).

**Fig. 4:**
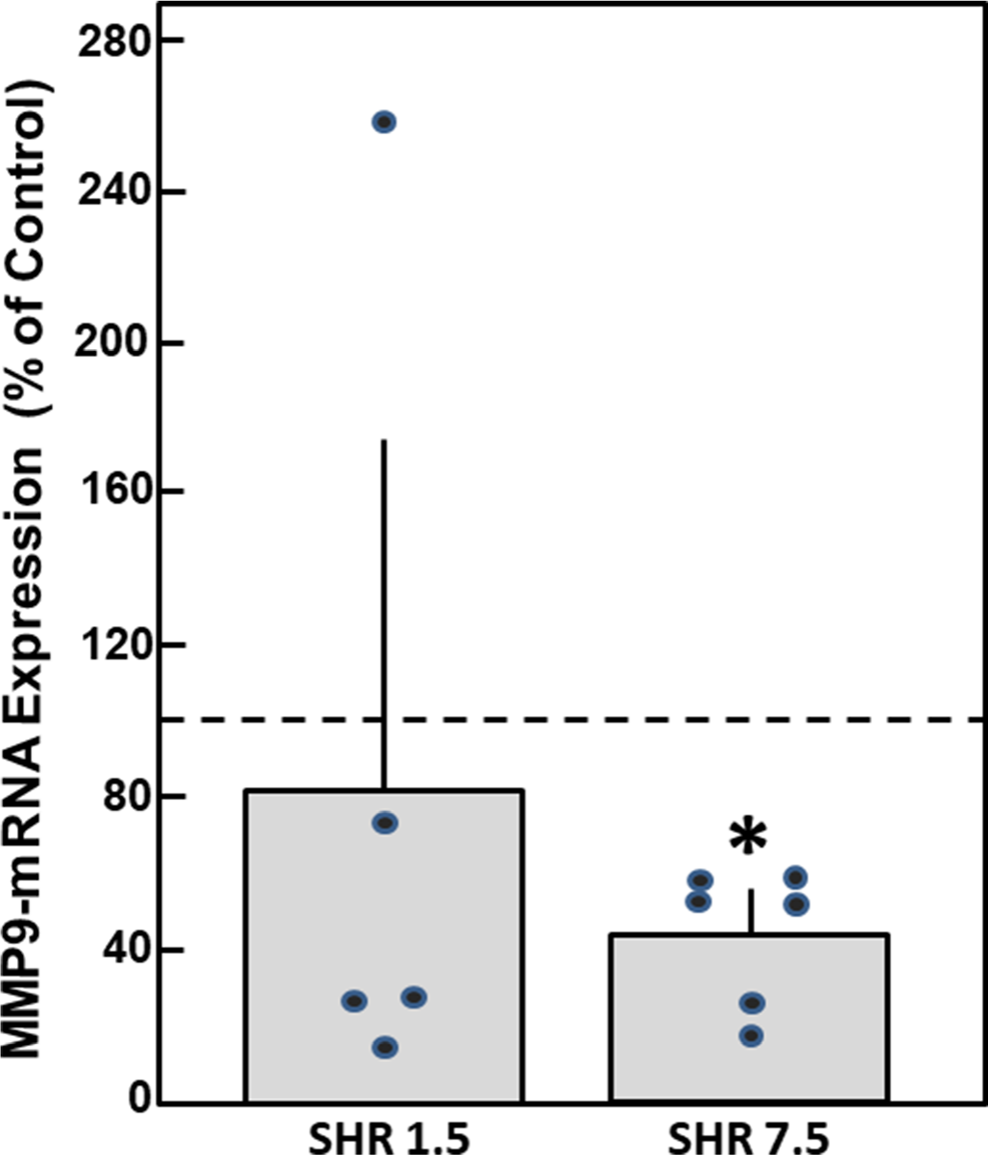
MMP mRNA expression in SHR rats. Total RNA was isolated from the hearts of pre-hypertensive 1.5-month-old and hypertensive 7.5-month-old SHR rats, as well as from age-matched normotensive Wistar rats. mRNA expression of MMP-9 was analyzed by real-time RT-PCR (*n* = 5 – 6, *p < 0.05 vs. normotensive Wistar rats)

In order to assess whether MMP downregulation can be observed in vivo, too, we analyzed MMP-2 and MMP-9 mRNA expression in spontaneous hypertensive rats (SHR). As published earlier, these animals are pre-hypertensive in the age of 1.5 months but develop a manifest high blood pressure at the age of 7.5 months, which goes along with cardiac hypertrophy, as determined by heart weight/tibia length (data published in [15]). The MMP mRNA expression in specimens of these animals revealed a decline in MMP-9 mRNA expression in 7.5-month-old SHR rats to 42 ± 15% compared to age-matched normotensive Wistar rats (*n* = 6, *p* < 0.05) (Fig. 4). MMP-2 mRNA levels remained unchanged (data not shown).

### MMP Inhibition Enhances Hypertrophic Growth of Cardiac Myocytes

**Fig. 5:**
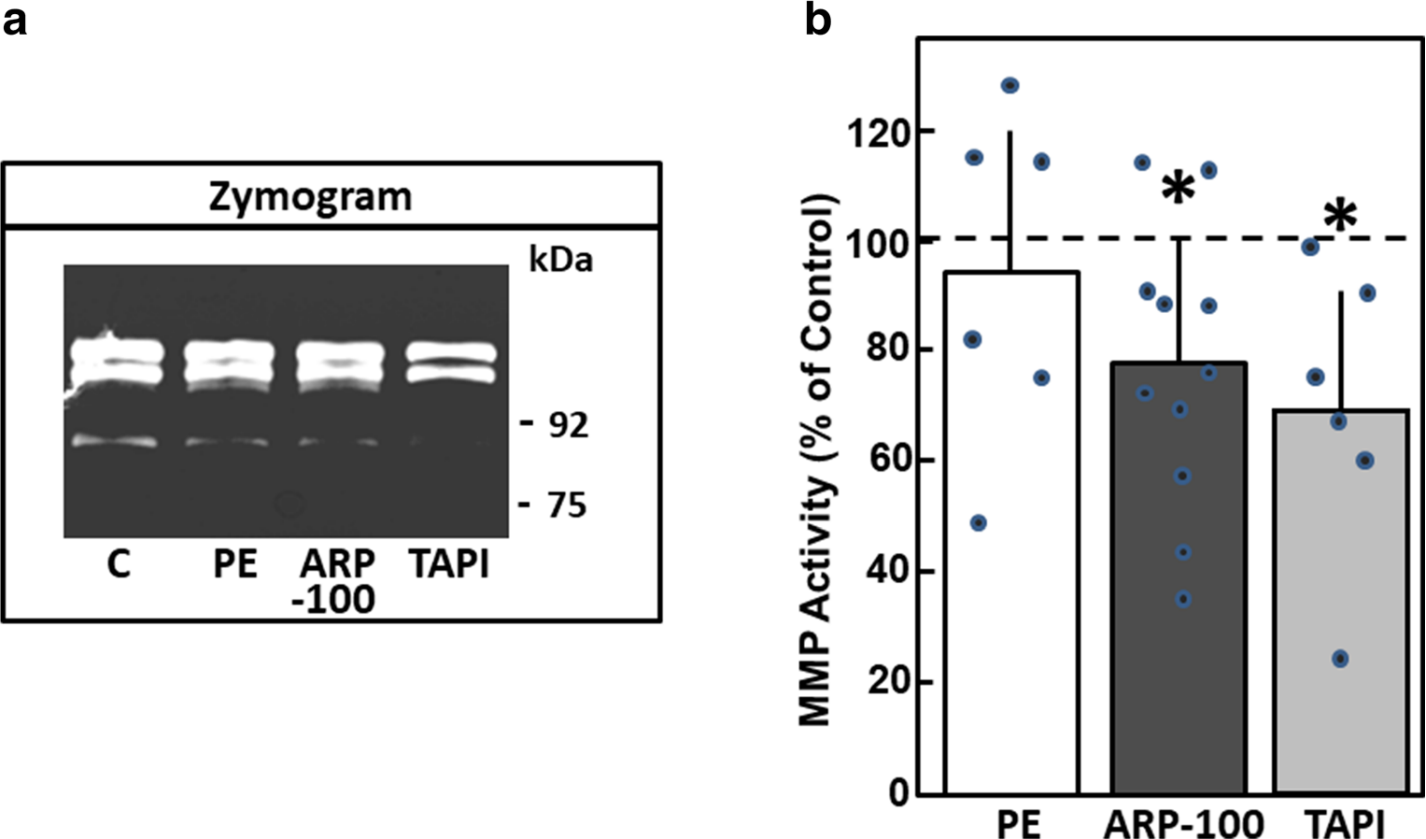
Pharmacologic inhibition of MMP activity. Cardiomyocytes were incubated with phenylephrine (PE, 10 μM), ARP-100 (50 nM), or TAPI-0 (50 μM) for 24 h. Then, MMP activity was determined by zymography. (A) Representative zymogram. (B) Densiometric evaluation of MMP activity (*n* = 6 for PE, *n* = 11 for ARP-100, *n* = 6 for TAPI-0, *p* < 0.05 vs. control)

The finding that MMP expression in cardiac myocytes is rather down- than upregulated under hypertrophic growth stimulation suggested that MMP attenuation might be involved in the process of hypertrophic growth. Hence, we tested the effect of different MMP inhibitors on hypertrophic growth. We used three different kinds of MMP inhibitors: TAPI-0 as a nonselective pharmacologic inhibitor known to inhibit MMP-1, MMP-2, MMP-8, MMP-9, MMP-13, and MMP-18; recombinant human tissue inhibitors of metalloproteinases type 2 (TIMP2), which also has a broad spectrum of MMP inhibition; and ARP-100 as a more specific pharmacological inhibitor of MMP-2. Reduction of the gelatinolytic activity of MMPs by the pharmacological inhibitors TAPI-0 and ARP-100 was verified in zymography assays (Fig.5). As depicted, TAPI-0 reduced total gelatinolytic activity to 70.0 ± 24.2% (*n* = 6, *p* < 0.05), and ARP-100 to 77.4 ± 24.4% compared to controls (*n* = 11, *p* < 0.05) (Fig. 5B).

**Fig. 6:**
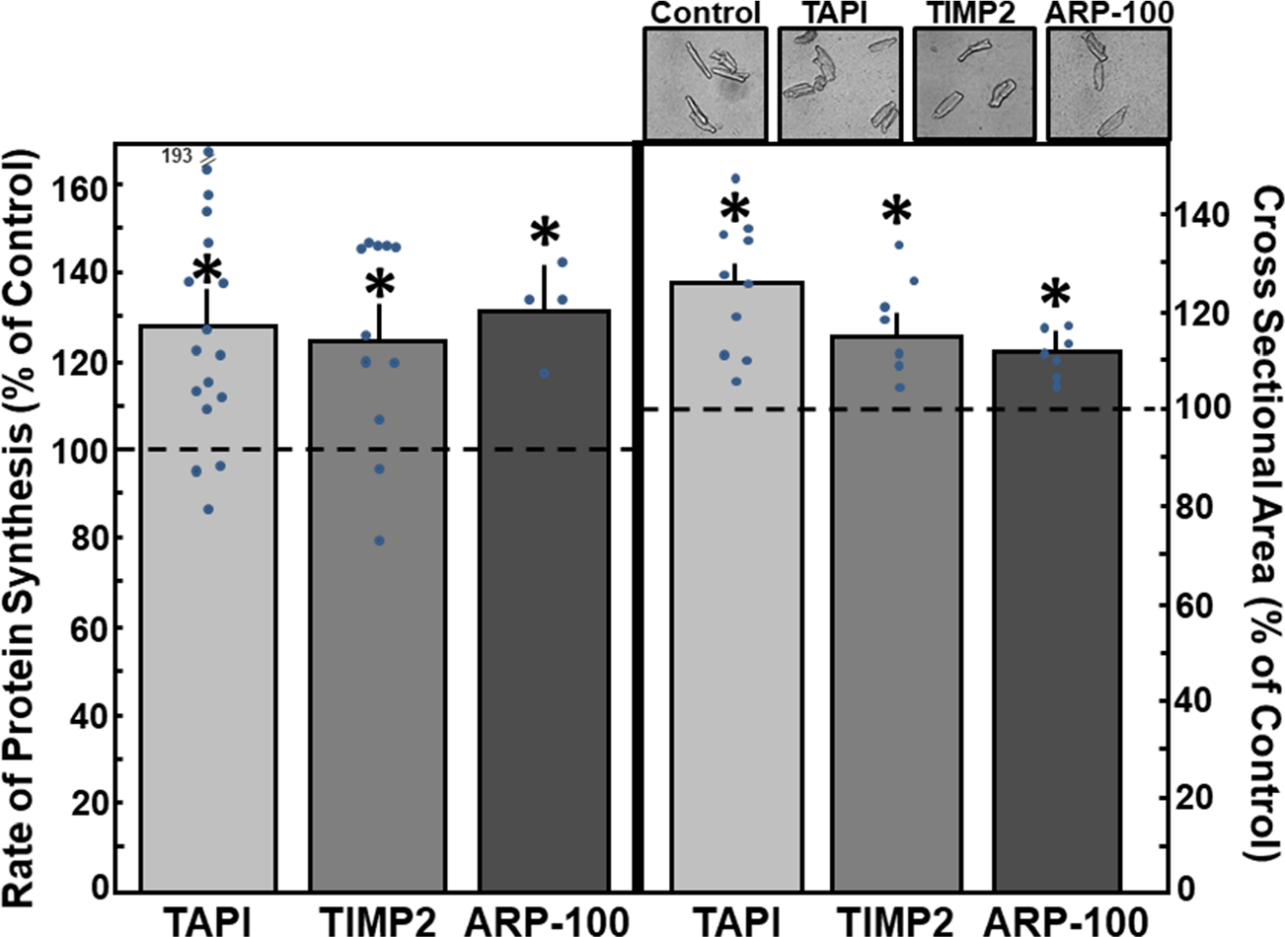
MMP inhibitors enhance hypertrophic growth. Cardiomyocytes were incubated with the MMP inhibitors TAPI-0 (50 μM), TIMP2 (24 mM), and ARP-100 (50 nM) for 24 h. For detection of hypertrophic growth, incorporation of ^14^C-phenylalanine during 24 h, or cross-sectional area, was determined after 24 h. Data are expressed as percent increase relative to untreated controls and are means ± SE of 4–17 independent culture preparations. *Differences from unstimulated controls with *p* < 0.05

Then, the effects of the inhibitors on cardiac myocytes hypertrophy were tested. Addition of 50 μM TAPI-0 to cardiac myocytes for 24 h increased the rate of protein synthesis to 128.9 ± 6.8% (*n* = 17, *p* < 0.05 vs. control) (Fig. 6) and an increase in cross-sectional area of the cardiomyocytes up to 125.6 ± 4.4% was found (297 cells in *n* = 10, *p* < 0.05 vs. control) (Fig. 6). Since TAPI-0 is also known as an inhibitor of TNFα-convertase, we used the TNFα antagonist WP9QY to exclude that the effects of TAPI-0 were due to the inhibition of TNFα signaling. Incubation of cardiomyocytes with WP9QY (25 μM) for 24 h did not enhance the rate of protein synthesis (102.8 ± 3.0%, *n* = 6, n.s. vs. control). Incubation of cardiac myocytes with the TIMP2 (24 mM) for 24 h increased the rate of protein synthesis to 124.3 ± 8.4% (*n* = 11, *p* < 0.05 vs. control) and cross-sectional area to 117.2 ± 3.3% (246 cells in *n* = 7, *p* < 0.05 vs. control) (Fig. 6). Furthermore, ARP-100 (50 nM) enhanced the rate of protein synthesis to 131.4 ± 10.0% (*n* = 4, *p* < 0.05), and the cross-sectional area of cardiac myocytes to 111.4 ± 4.2% (277 cells in *n* = 7, *p* < 0.05) (Fig. 6). Thus, independent of the specificity of the MMP inhibitor, reduction of MMP activity in cardiac myocytes provoked hypertrophic growth.

### Hypertrophic Signaling Under MMP Inhibition

To analyze if extracellular matrix modifications have contributed the hypertrophic growth responses under MMP inhibition, we determined integrin1β expression under TAPI-0 or ARP-100. However, integrin1β expression did not change, neither on the mRNA nor on the protein level (mRNA: 1.01 ± 0.4 times under TAPI-0 and 1.04 ± 0.5 times under ARP-100, *n* = 7, n.s. vs. control; and protein: 99 ± 33% under TAPI-0, and 119 ± 56 under ARP-100, *n* = 7, n.s. vs. control).

Cardiac hypertrophy can be induced by diverse factors and conditions that ultimately converge on a limited number of protein kinases. Among others, ERK, PI3K (phosphatidylinositol-3-kinase), and Akt are prominent kinases, most often found to be involved in hypertrophic signaling [29]. Therefore, we tested the involvement of these kinases in hypertrophic growth stimulation following MMP inhibition by TAPI-0.

**Fig. 7:**
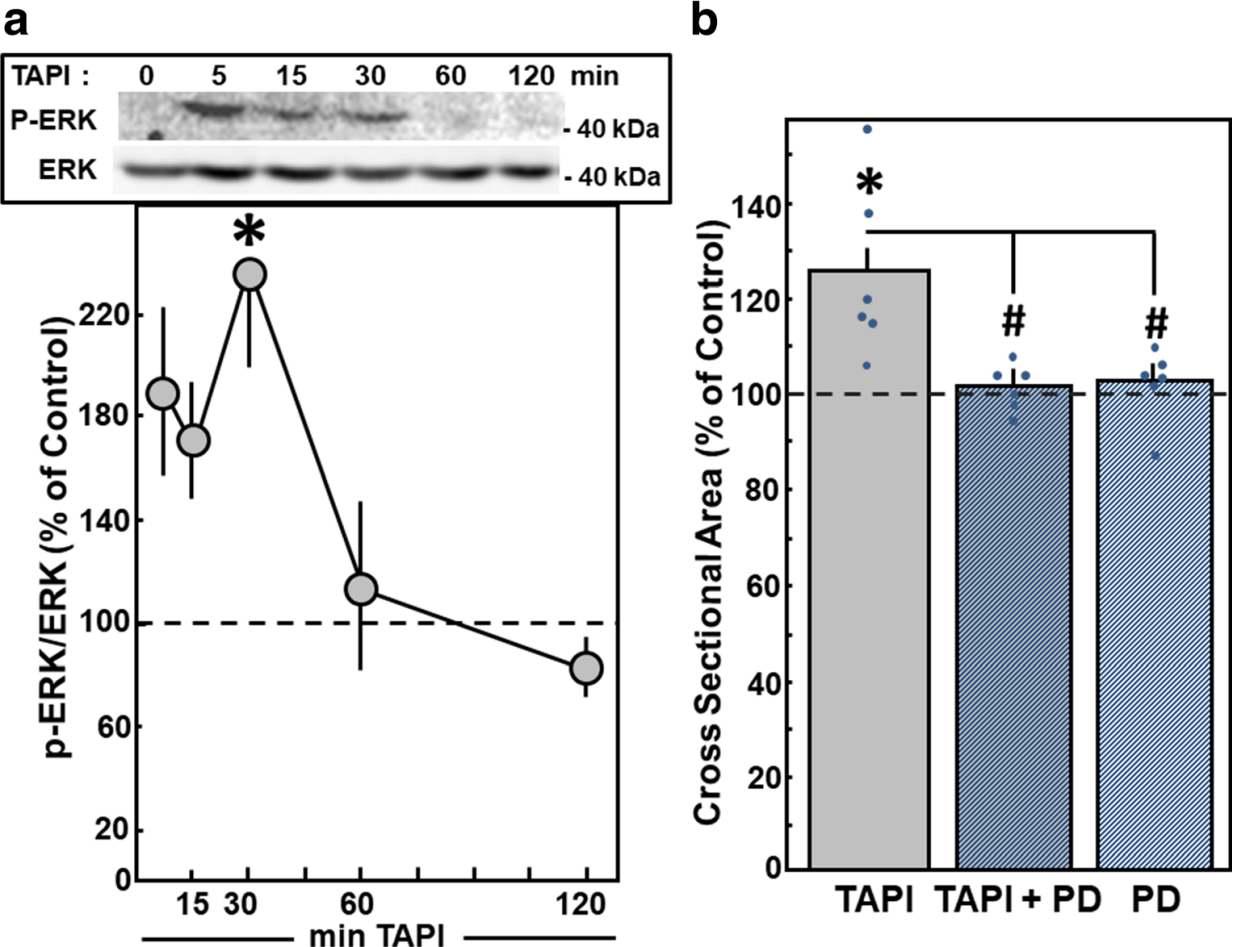
Hypertrophic growth under MMP inhibition is mediated by ERK. Cardiomyocytes were stimulated with TAPI-0 (50 μM) for 24 h. For inhibition of ERK, PD98059 (PD, 10 μM) was added 30 min before TAPI-0. (A) For detection of phosphorylated and, thus, activated ERK, cardiomyocytes were stimulated with TAPI-0 (50 ng/ml) for 5 min up to 2 h. Total protein extracts were prepared, separated on 10% SDS gels. ERK activation was determined in Western blots by determination of p-ERK/ERK levels. Data are expressed as percent increase relative to untreated controls and are means ± SE of seven independent culture preparations. *Differences from unstimulated controls with *p* < 0.05. (B) For detection of hypertrophic growth, an increase in cross-sectional area during 24 h was determined. Data are expressed as percent increase relative to untreated controls and are means ± SE of six independent culture preparations. *Differences from unstimulated controls with *p* < 0.05. ^#^Differences from TAPI-0 stimulated cells with *p* < 0.05

As a sign for ERK activation, phosphorylation of ERK was determined in Western blots. After addition of TAPI-0, ERK activation was visible within 5 min. A significant enhancement to 238.9 ± 38.4% was detected after 30 min (*n* = 7, *p* < 0.05 vs. control). Thereafter, ERK activity declined to baseline levels (Fig. 7A). Involvement of ERK in TAPI-0-induced hypertrophy was determined by the inhibition of ERK signaling with 10 μM PD98059, which is a highly selective inhibitor of MEK1 and the MAP kinase cascade. ERK inhibition abolished TAPI-0-induced hypertrophic growth, since cell size did not increase under TAPI-0 in the presence of PD98059 (100.8 ± 4.3%, *n* = 6, *p* < 0.05 vs. TAPI-0 stimulation) (Fig. 7B). PD98059 per se did not modify cross-sectional area.

**Fig. 8:**
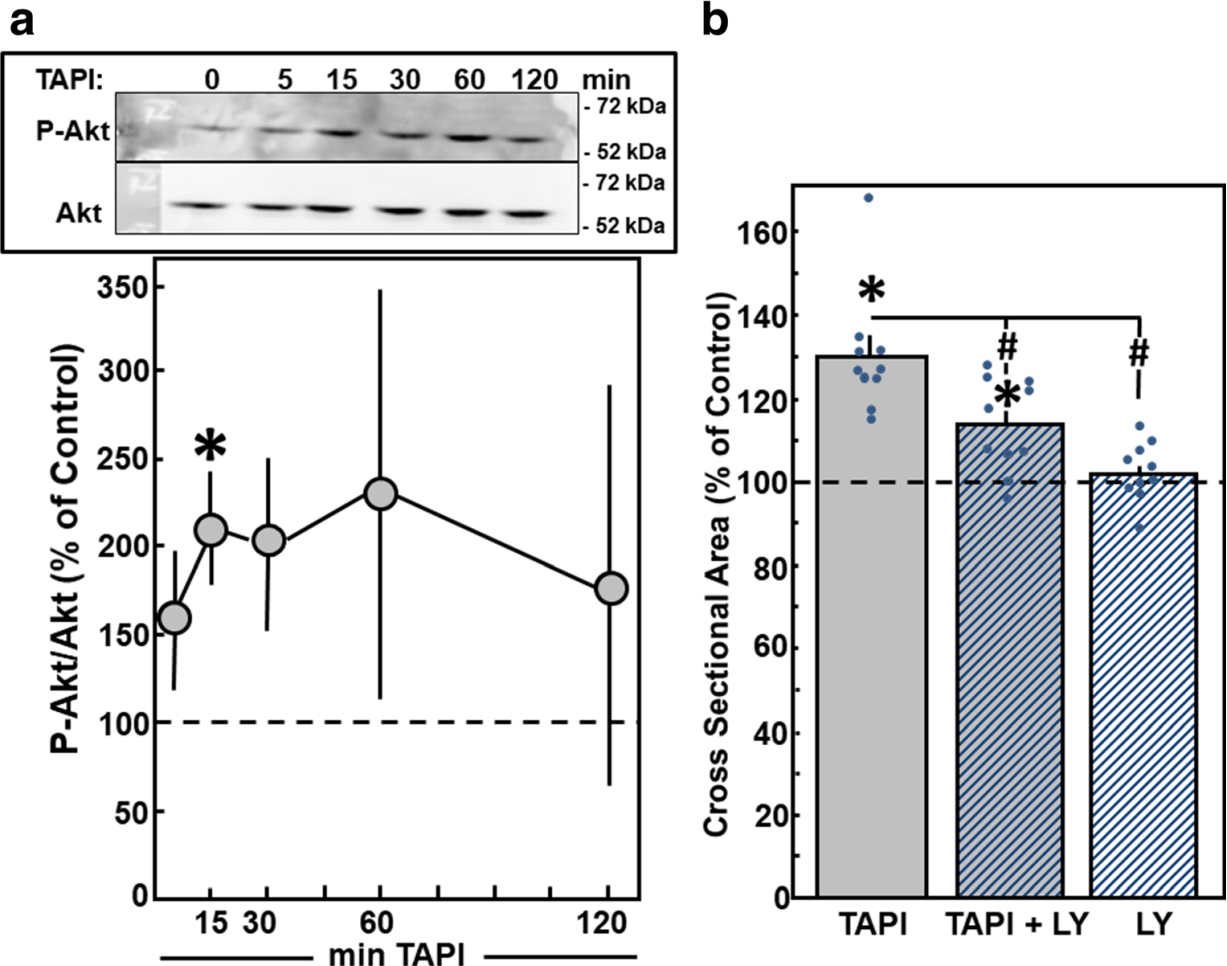
Hypertrophic growth under MMP inhibition is mediated by PI3K. Cardiomyocytes were stimulated with TAPI-0 (50 μM) for 24 h. For inhibition of PI3K, LY290042 (LY, 10 μM) was added 30 min before TAPI-0. (A) For detection of phosphorylated and thus activated PI3K, cardiomyocytes were stimulated with TAPI-0 (50 ng/ml) for 5 min up to 2 h. Total cell extracts were prepared and separated on 10% SDS-gels. PI3K activation was determined in Western blots by determination of p-PI3K. (B) For detection of hypertrophic growth, an increase in cross-sectional area during 24 h was determined. Data are expressed as percent increase relative to untreated controls and are means ± SE of *n* = 4–10 independent culture preparations. *Differences from unstimulated controls with *p* < 0.05. ^#^Differences from TAPI-0 stimulated cells with *p* < 0.05

As indicator for activation of PI3K/Akt-signaling, phosphorylation of Akt was detected in Western blots within 15 min after addition of TAPI-0 (Fig. 8A). Incubation of cardiac myocytes with LY290042 (10 μM) for 30 min prior to TAPI-0 stimulation reduced the hypertrophic growth response. The cross-sectional area of cardiomyocytes under TAPI-0 stimulation did not increase in the presence of LY290042 (113.7 ± 3.6%, *n* = 10, *p* < 0.05 vs. TAPI-0 stimulation, n.s. vs. control) (Fig. 8B). LY290042 per se did not modify the cross-sectional area.

A classical downstream target of PI3K in hypertrophic signaling is the inhibition of GSK3β (glycogen synthase3 β). GSK3β is constitutively expressed in cardiac myocytes and was among the first negative regulators of cardiac hypertrophy to be identified [30, 31]. Similarly, here we identified MMPs as negative regulators of cardiomyocytes growth. We now analyzed if inhibition of MMPs may also inactivate GSK3β and if it may promote hypertrophic growth via this pathway.

**Fig. 9:**
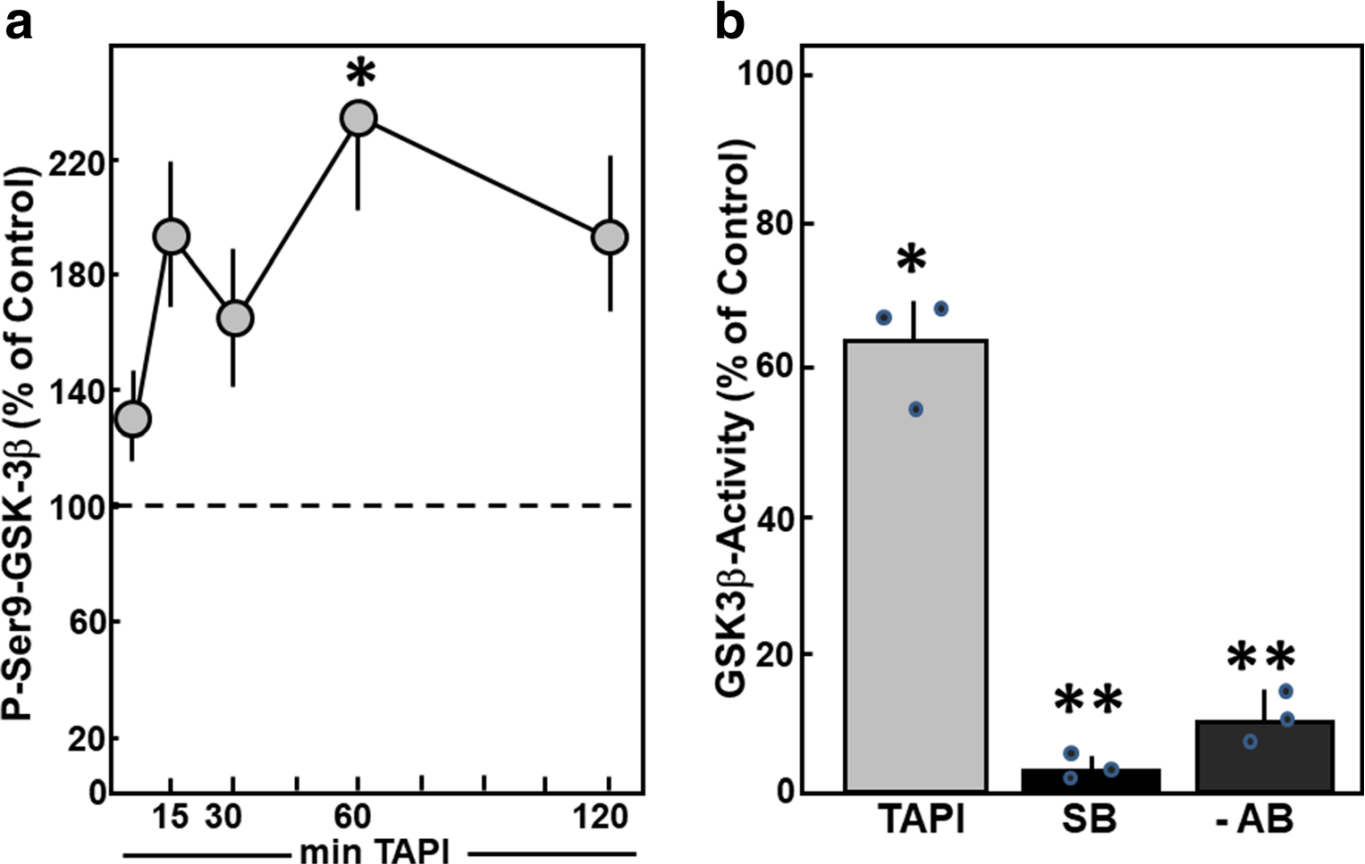
MMP inhibition reduces GSK3β phosphorylation and activity. (A) Cardiomyocytes were stimulated with TAPI-0 (50 ng/ml) for 5 min up to 2 h. Total cell extracts were prepared and separated on 10% SDS gels. GSK3β activation was determined in Western blots using antibodies specific for P-serine9-GSK3β. Data are expressed as percent increase relative to untreated controls and are means ± SE of nine independent culture preparations. *Differences from unstimulated controls with *p* < 0.05. (B) Cardiomyocytes were stimulated with TAPI-0 (50 ng/ml) for 1 h. Total cell extracts were prepared, GSK3β was immunoprecipitated, and phosphorylation of GSK3β substrate peptide was determined in vitro. Controls for GSK3β specificity were done either by addition of the GSK3β inhibitor SB 415286 (SB) to the kinase reaction or by omission of GSK3β antibodies (AB) during immunoprecipitation. Data are expressed as percent increase relative to untreated controls and are means ± SE of three independent culture preparations. *Differences from unstimulated controls with *p* < 0.05. **Differences from unstimulated controls with *p* < 0.01

Indeed, Ser9 phosphorylation of GSK3β, which is a sign of GSK3β inactivation, was enhanced after addition of TAPI-0. Phosphorylation at Ser9 reached a maximum 60 min after addition of TAPI-0 (236.7 ± 33.1%, *n* = 9, *p* < 0.05 vs. control) (Fig. 9A). To confirm these findings of GSK3β inactivation under MMP inhibition, the activity of immunoprecipitated GSK3β was determined by incorporation of ^32^P in a substrate peptide in vitro. One hour after addition of TAPI-0, a reduction of GSK3β activity to 62.3 ± 4.2% was detected (*n* = 3, *p* < 0.05 vs. control) (Fig. 9B). The addition of the GSK3β inhibitor SB415286 to the kinase reaction abolished GSK3β activity. As well, GSK3β activity was absent when immunoprecipitation was performed in the absence of GSK3β antibodies. These controls confirm the specificity of the GSK3β activity assay.

## Discussion

The main finding of the study is the identification of MMPs as repressors of hypertrophic growth in cardiomyocytes. This could be demonstrated by the enhancement of the rate of protein synthesis and of cardiomyocytes’ size under nonspecific inhibition of MMPs and inhibition of MMP-2.

As could be shown in PCR and Western blots, different MMP types are expressed in isolated cardiomyocytes of adult rat, namely, MMP-1, MMP-2, MMP-3, MMP-9, and MMP-14. Of these transcripts, only MMP-2, MMP-9, and MMP-14 are sufficiently translated into proteins, so that they were detected by Western blot. The reasons for non-detection of MMP-1 and MMP-3 on the protein level may be a non-efficient translation of the mRNA or a strong secretion of these MMP types into the medium so that they cannot be detected in protein extracts of the cardiomyocytes. Furthermore, as we did not use a positive control in Western blots, we cannot completely exclude that the antibodies were working insufficiently. In spite of this limitation, we can conclude that the expression profile of MMPs in cardiomyocytes is clearly reduced compared to the MMP types found in myocardium (reviewed by Spinale [22]), thereby indicating that other cell types in the heart, like endothelial cells or fibroblasts, are the source of the greater MMP diversity in the myocardium.

Interestingly, we did not observe any stimulation of MMPs under pro-hypertrophic treatment. However, mRNA downregulation of MMP-1, MMP-9, or MMP-14 under stimulation of cardiomyocyte hypertrophy by phenylephrine or AngII, as well as under TGFβ_1_ stimulation, was detected, and MMP-9 was reduced by phenylephrine and AngII. Different subsets of MMPs were downregulated by each of the hypertrophic stimuli. Reasons for this may rely on the different receptors that transfer the hypertrophic stimulus into the cell (adrenoreceptor, AT1-receptor, or TGFβ receptor), which are coupled to diverse signaling cascades. For example, the TGFβ_1_ receptor is tightly coupled to transcription factors of the SMAD family [32], while phenylephrine signals via basic leucine zipper (bZIP) family of transcription factors, like AP-1 [33], thereby conveying different promotor specificities. In hypertensive SHR rats which developed cardiac hypertrophy, we also observed downregulation of MMP-9 mRNA while MMP-2 mRNA was preserved. This fits quite well to our observation that MMP-9 is downregulated under adrenoceptor stimulation in cardiomyocytes. In contrast to our observations of downregulation of MMPs, in animal models with induction of cardiac hypertrophy, MMPs are often found to be upregulated, i.e., under AngII infusion, induction of MMP-7 was shown [34]; under norepinephrine infusion [35] or pressure overload [8], MMP-2 was induced. In MMP-2 knockout mice cardiac hypertrophy under pressure overload was repressed [8]. There are several reasons that may contribute to these differences: (1) regulation of MMPs may be stimulus dependent, (2) time dependent, (3) MMP-type dependent, and (4) source dependent. The sources of MMPs in animal studies were not determined and might be different from cardiomyocytes. The release of MMPs by, e.g., fibroblasts may preferably contribute to extracellular matrix remodeling and fibroblast proliferation, which then is a trigger for hypertrophic growth. Furthermore, in the heart, mechanical stress may, in addition to the stimuli used in our in vitro study, influence MMP expression [36]. Thus, we clearly have to distinguish between the anti-hypertrophic action of MMPs in cardiomyocytes and pro-hypertrophic actions by extracellular matrix remodeling of heart tissue. In future studies, co-culture systems should be applied in order to analyze the mutual influence of cardiomyocytes and fibroblasts.

Downregulation of MMPs under hypertrophic growth stimulation in cardiomyocytes indicated that MMP downregulation could be functionally involved in the hypertrophic growth process of cardiomyocytes. Indeed, we show that MMP inhibitors induce hypertrophic growth in isolated cardiomyocytes, independent of other additional hypertrophic stimuli. This indicates that the presence of MMPs under baseline conditions reduces hypertrophic growth of cardiomyocytes. These findings are similar to the induction of left ventricular hypertrophy in EMMPRIN knockout mice [37]. EMMPRIN is an endogenous stimulator of MMPs. In EMMPRIN knockout mice, reduced MMP-1 and MT1-MMP levels were found. Aberrant extracellular matrix remodeling occurred in these animals that contributed to ventricular hypertrophy. If cardiomyocyte growth was also involved in the hypertrophic growth process, it was not analyzed in that study. However, since we did not detect MMP-1 in cardiomyocytes, the development of cardiac hypertrophy in EMMPRIN knockout mice might differ from that in isolated cardiomyocytes.

In our study, MMP inhibitors with a nonselective MMP inhibition (TAPI-0 and TIMP2) were used, so that we cannot attribute the anti-hypertrophic role to one specific MMP type. But in zymography, efficient repression of MMP activity by TAPI-0 was proven. Furthermore, the more specific MMP-2 inhibitor ARP-100 also induced hypertrophic growth, thereby indicating that MMP-2 is at least one of the anti-hypertrophic MMPs found in cardiomyocytes. ARP-100 was used as MMP-2 specific inhibitor already in other studies on heart disease: in isolated hearts, ARP-100 reduced ischemia-reperfusion injury via protection against junctophilin 2 or SERCA (Sarcoplasmic reticulum calcium ATPase) degradation [38, 39]. Thus, ARP-100 promotes cardiomyocyte hypertrophy but prevents contractile dysfunction in ischemic reperfused myocardium. These divergent findings imply protective as well as detrimental roles of MMP-2 in the heart.

The reduction of only 20–30% of MMP activity by TAPI-0 or ARP-100 affects signaling and promotes phenotypic changes in cells. However, given the fact that nonselective pharmacological inhibitors were used in the present study, we are unable to draw solid conclusions on the MMPs involved in our findings. In future studies, more specific and efficient inhibition of MMPs, e.g., via siRNA approaches, should be applied in order to shed light on the multiple functions of MMPs in cardiomyocytes.

Other studies that revealed anti-hypertrophic actions of MMPs were in vivo studies in mice under hemodynamic stress, induced either by AngII infusion or transaortic constriction (TAC). In ADAM22 [40], as well as in ADAM12 knockout mice [41], cardiac hypertrophy was aggravated under pressure overload. In both knockout animals, enhancement of ERK or Akt signaling was seen that may have contributed to cardiac hypertrophy. Also in our study, we observe activation of ERK and Akt under MMP inhibition. By the use of specific inhibitors against ERK and PI3K, which is a direct activator of Akt [42], we now demonstrate the involvement of these kinases in hypertrophic growth under MMP inhibition. Our data imply that also in ADAM22 and 12, ERK/Akt signaling may be involved in the hypertrophic growth process.

Mechanistically, Akt1 directly promotes protein translation, in part by inhibiting GSK3β activity, which negatively regulates eukaryotic translation initiation factor 2B and thereby represses cardiac hypertrophy [30]. In our study, we present evidence that MMP inhibition represses GSK3β activity in cardiomyocytes. Therefore, via inhibition of the repressor function of GSK3β, MMP inhibition might promote the hypertrophic growth response in cardiomyocytes.

Thus, induction of intracellular processes upon deletion or inhibition of MMPs can be observed in animal models and in isolated cardiomyocytes. These results raise the question of how such an intracellular activation can be achieved by extracellular matrix remodeling enzymes. Two possible explanations are conceivable: (i) MMPs act via extracellular modulation that is transferred into the cell or (ii) MMPs possess a direct intercellular action, because they are located at nearly all intracellular structures in cardiomyocytes, i.e., at the sarcomer [43], in the nucleus [44], in mitochondria [45], caveolae-bound [46], and in the cytosol [47].

The extracellular action of MMPs can modulate cell surface receptors on cardiomyocytes that transfer information into the cell. ADAM22, for example, interacts with integrin-linked kinase which targets PI3K [48]. Loss of ADAM12 or 17 mediated cardiac hypertrophy via increased integrin B1 levels that result in overactivation of integrin-FAK signaling. FAK (Focal adhesion kinase) directly associates and activates PI3K and induces hypertrophic signaling via this pathway [49]. Also we identified involvement of PI3K and ERK in hypertrophic growth under MMP inhibition in cardiomyocytes. However, in our study, an increased expression of integrin1β was not observed under MMP inhibition by TAPI-0 or ARP-100. Therefore, the integrin pathway found in ADAM knockout mice, which acts via modulation of the extracellular surface of cardiomyocytes, may not be responsible for hypertrophic growth effects under MMP inhibition in cardiomyocyte.

Thus, the second possibility of MMP action, namely, a direct intracellular action mechanism, may be responsible for the hypertrophic action under MMP inhibition. As already mentioned above, MMPs are found localized in cardiomyocytes at sarcomeric structures, the nucleus, and mitochondria and may thus directly repress hypertrophic signaling in the cell. However, up to now there are no studies that presented evidence for the involvement of intracellular localized MMPs in hypertrophic growth.

**Fig. 10:**
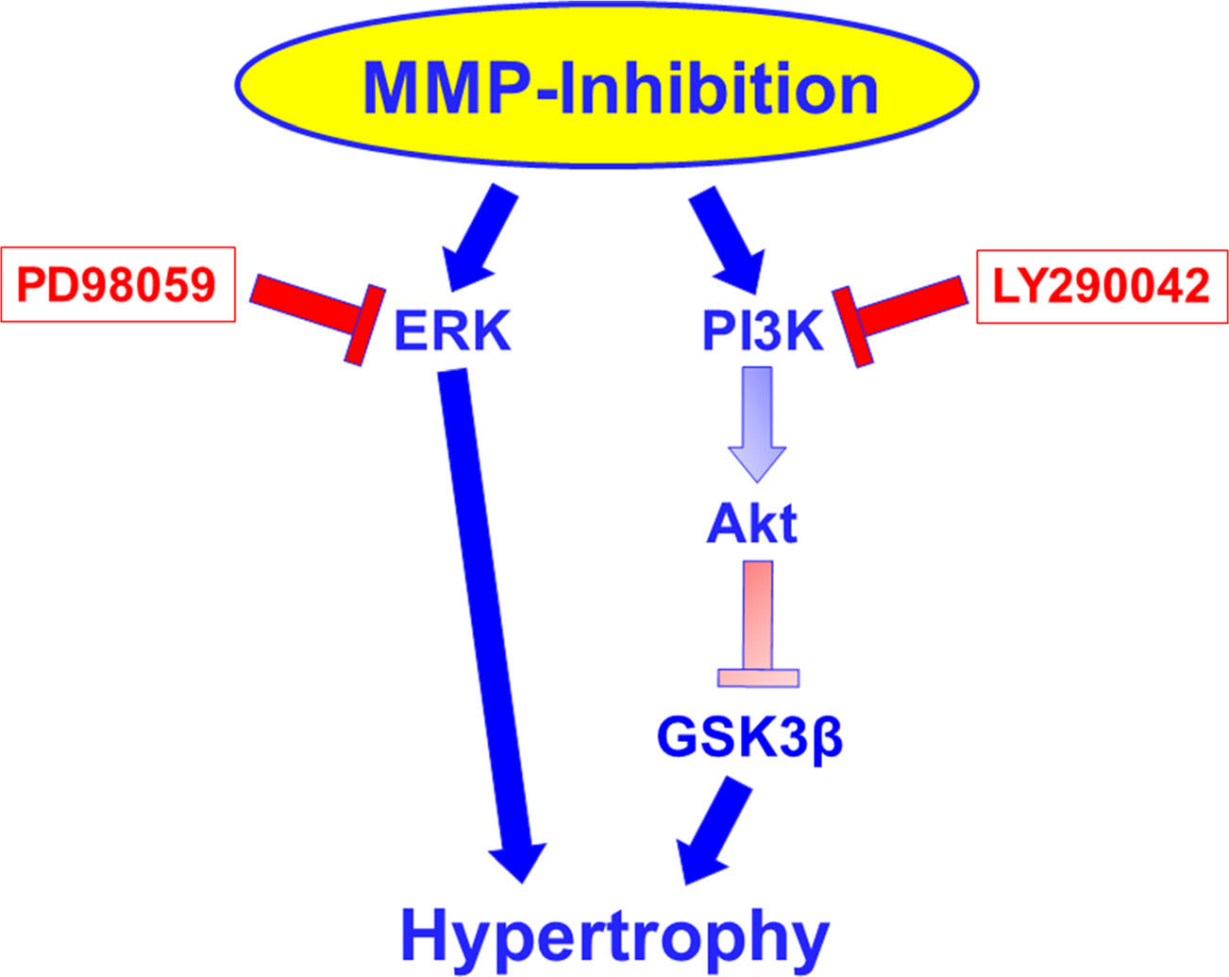
Proposed signaling pathway under MMP inhibition. Inhibitors used for dissection of the pathway are indicated in red rectangles. The pathway via Akt and GSK3 beta is assumed due to the activation/inhibition of these molecules under MMP inhibition combined with the knowledge that PI3K can signal via these pathways, as outlined in the discussion

In conclusion, MMPs found in cardiomyocyte culture may act as repressor of cardiomyocyte hypertrophy under baseline conditions. Active forms of MMPs are expressed in cardiomyocytes, and inhibition of them promotes hypertrophic growth via ERK and PI3K-GSK3β signaling (Fig. 10). Thus, here we characterized a new aspect of MMP function in cardiomyocytes. Therefore, MMPs in the healthy heart may be important players to repress cardiac hypertrophy.

## Data Availability

The data that support the findings of this study are available from the corresponding author upon reasonable request.
